# Serum microRNA profiles in athyroid patients on and off levothyroxine therapy

**DOI:** 10.1371/journal.pone.0194259

**Published:** 2018-04-12

**Authors:** Elske T. Massolt, Layal Chaker, Theo J. Visser, Ad J. M. Gillis, Lambert C. J. Dorssers, Carolien M. Beukhof, Boen L. R. Kam, Gaston J. Franssen, Giulia Brigante, Tessa M. van Ginhoven, W. Edward Visser, Leendert H. J. Looijenga, Robin P. Peeters

**Affiliations:** 1 Division of Endocrinology, Erasmus MC, Rotterdam, The Netherlands; 2 Academic Center for Thyroid Diseases, Department of Internal Medicine, Erasmus MC, Rotterdam, The Netherlands; 3 Department of Epidemiology, Erasmus MC, Rotterdam, The Netherlands; 4 Department of Pathology, Erasmus MC, Rotterdam, The Netherlands; 5 Department of Nuclear Medicine, Erasmus MC, Rotterdam, The Netherlands; 6 Division of Surgical Oncology, Department of Surgery, Erasmus MC Cancer Institute, Rotterdam, The Netherlands; 7 Unit of Endocrinology, Department of Biomedical, Metabolic and Neural Sciences, University of Modena and Reggio Emilia, Modena, Italy; Universidade do Porto Faculdade de Medicina, PORTUGAL

## Abstract

**Background:**

Levothyroxine replacement treatment in hypothyroidism is unable to restore physiological thyroxine and triiodothyronine concentrations in serum and tissues completely. Normal serum thyroid stimulating hormone (TSH) concentrations reflect only pituitary euthyroidism and, therefore, novel biomarkers representing tissue-specific thyroid state are needed. MicroRNAs (miRNAs), small non-coding regulatory RNAs, exhibit tissue-specific expression patterns and can be detectable in serum. Previous studies have demonstrated differential expression of (precursors of) miRNAs in tissues under the influence of thyroid hormone.

**Objective:**

To study if serum miRNA profiles are changed in different thyroid states.

**Design and methods:**

We studied 13 athyroid patients (6 males) during TSH suppressive therapy and after 4 weeks of thyroid hormone withdrawal. A magnetic bead capture system was used to isolate 384 defined miRNAs from serum. Subsequently, the TaqMan Array Card 3.0 platform was used for profiling after individual target amplification.

**Results:**

Mean age of the subjects was 44.0 years (range 20–61 years). Median TSH levels were 88.9 mU/l during levothyroxine withdrawal and 0.006 mU/l during LT4 treatment with a median dosage of 2.1 μg/kg. After normalization to allow inter-sample analysis, a paired analysis did not demonstrate a significant difference in expression of any of the 384 miRNAs analyzed on and off LT4 treatment.

**Conclusion:**

Although we previously showed an up-regulation of pri-miRNAs 133b and 206 in hypothyroid state in skeletal muscle, the present study does not supply evidence that thyroid state also affects serum miRNAs in humans.

## Introduction

Hypothyroidism is one of the most common endocrine disorders [1]. In a subset (~10–15%) of patients, symptoms of hypothyroidism persist despite serum thyroid hormone concentrations within the laboratory reference range during levothyroxine (LT4) replacement therapy [2–4]. One of the explanations for this impaired well-being might be the inadequacy of LT4 treatment to restore physiological thyroxine (T4) and triiodothyronine (T3) concentrations, especially the T4/T3 ratio, in serum and tissues [5, 6]. Studies in hypothyroid rats showed that LT4 monotherapy was unable to normalize concentrations of T4 and T3 in all tissues [7]. Supra-physiological serum T4 concentrations had to be reached in most tissues to normalize tissue T3 concentrations and the LT4 dose required to normalize thyroid hormone concentrations was different for each tissue.

Serum TSH concentrations, reflecting the pituitary feedback to thyroid hormone, is used in clinical practice to monitor LT4 treatment because it is the best available marker of thyroid state. However, although TSH typically reflects local T3 concentrations in the pituitary, it may not necessarily reflect local thyroid status in all tissues, especially when thyroid hormone production is not endogenously controlled, such as in athyreotic patients during LT4 therapy [8, 9]. For this reason, novel markers representing thyroid state of other tissues than the pituitary would be of great clinical relevance.

MicroRNAs (miRNAs) are non-coding RNA molecules with a length of approximately 22 nucleotides, which predominantly post-transcriptionally repress the translation of mRNAs from target genes by binding to the 3’UTR of messenger RNA [10–12]. Overall, miRNAs exhibit tissue-specific expression patterns and each miRNA may affect the expression of hundreds of target genes. Recently, we reported on gene expression profiles in skeletal muscle of hypothyroid patients off and on LT4 therapy and found a large up-regulation in expression of the muscle-specific pri-miRNAs 133b and 206 in hypothyroid state [13]. This was supported by other studies that found an increase in expression of miRNAs- 1, 206, 133a and 133b in livers of hypothyroid mice compared to euthyroid controls [14]. MiRNAs can also be present in the circulation and have been associated with a variety of diseases [15, 16]. We therefore hypothesized that miRNA profiles in serum can also be influenced by thyroid state, and that miRNAs derived from different tissues potentially reflect tissue-specific differences in thyroid states.

## Materials and methods

### Patients

Patients with differentiated thyroid cancer (DTC) are exposed to different thyroid states as part of their treatment. These patients are subject to severe hypothyroidism before radioactive iodine (RAI) therapy to stimulate radioactive iodine uptake by malignant tissues, whereas they have relatively high thyroid hormone concentrations afterwards when receiving substitution therapy with LT4 to suppress TSH. As a consequence, these patients are an ideal model to study the consequences of different thyroid states longitudinally. Therefore, we used patients with DTC as a model to quantify serum levels of 384 miRNAs in different thyroid states. Since we were previously able to detect significant changes in numerous gene transcripts in peripheral blood as well as in muscle samples in respectively 8 and 10 patients, while using a similar study design, we postulated that 13 patients would be enough to identify clinically relevant markers [13, 17]. Using alpha = 0.05 and power = 80%, we would be able to detect a Cohen’s d effect size of 0.87, which is considered as a large effect.

13 DTC patients were consecutively recruited from the outpatient clinic of the Erasmus Medical Center, between May 1^st^ 2013 and February 1^st^ 2015. Patients were eligible for inclusion if they needed RAI therapy during LT4 withdrawal according to the Dutch guidelines [18], had no other malignancies or an active inflammatory disease, and were between 18 and 80 years old.

The study protocol was approved by the Medical Ethics Committee of the Erasmus Medical Center (MEC 2012–561) and written informed consent was obtained from all study participants.

### Thyroid function measurements

Peripheral blood samples were obtained in non-fasting conditions from all participants on and off LT4 treatment. Serum Free T4 (FT4, reference range 11–25 pmol/L) and total T3 (reference range 1.4–2.5 nmol/L) concentrations were measured by chemoluminescence assays (Vitros ECI Immunodiagnostic System; Ortho-Clinical Diagnostics, Rochester, MI). Serum TSH concentration (reference range 0.4–4.3 mU/L) was determined by immunometric assay (Immulite 2000 XPi, Siemens, Den Haag, the Netherlands). Serum samples were stored at −80 °C until further analysis of miRNAs.

### miRNA isolation from serum

The method of miRNA isolation and quantification has been previously described extensively [19]. In short, the miRNAs were purified from serum samples using TaqMan ABC Purification Kit—Human Panel A (Thermo Fisher, PN 4473087). These panels consist of superparamagnetic Dynabeads covalently bound to a unique set of 384 anti-miRNA oligonucleotides. The miRNAs match the miRNAs in Megaplex Pool described below. The panel includes exogenous and endogenous controls. Briefly, 100 μL of lysis buffer was added to 50 μL of serum. One μL of 1 nM of a non-human external control (ath-miRNA-159a) was added to monitor the extraction process, followed by the addition of 80 μL of beads (80 × 10^6^ beads). The tubes were mixed for 40 min at 1200 rpm and 30 °C, the beads were isolated using a magnetic bead separator, and washed three times with wash buffer. The bound miRNAs were eluted from the beads with 100 μL elution buffer and incubated at 70 °C for 3 min. The eluted miRNA pool was stored at −80 °C until ready to use.

For miRNA profiling, Megaplex Primer Pool A was used in conjunction with the matching TaqMan miRNA Array Card A. All reagents were purchased from Thermo Fisher/Life Technologies (Bleiswijk, NL). Briefly, 3 μL of the miRNA sample isolated with the ABC kit was reverse transcribed with the Megaplex RT Primer Pool A (PN 4399966) in a 8 μL final volume. The RT reaction was performed under thermal cycling (2 min at 16 °C, 1 min at 42 °C, 1 sec at 50 °C, for 40 cycles) and the enzyme was inactivated by treatment for 5 min at 85 °C. Four μL RT reaction was combined with its matching Megaplex PreAmp Primer Pool (PN 4399233) and TaqMan PreAmp Master Mix (PN 4391128) in a final volume of 25 μL. Pre-amplification was done using the following cycling conditions: 10 min at 95 °C; 2 min at 55 °C; 2 min at 72 °C; 15 sec at 95 °C, 4 min at 60 °C for 12 cycles; 99 °C for 10 min. The final pre-amplification product was diluted 1:100 in 1X TaqMan Universal Master Mix (PN 4364341), then loaded onto the matching TaqMan MiRNA Array Card A (PN 4398965) and run on a TaqMan^®^ 7900HT Fast Real-Time PCR System under universal cycling conditions.

### miRNA quantification

Raw data files were imported and analyzed using ExpressionSuite v1.0.4 (Life Technologies, South San Francisco, CA, USA), a software data analysis tool that can easily import and analyze large raw data files. In these experiments, the quantification cycle (Cq) is defined as the fractional cycle at which the amplification plot crosses the fluorescence threshold (Ct). The baseline was set automatically and the threshold was manually set at 0.2 and adjusted whenever appropriate to get an intersection in the exponential part of the curve. To capture as many differentially expressed miRNAs as possible, the threshold was set at 40 instead of 30–32 which is generally recommended by Life Technologies for relatively high miRNA levels. Undetermined values were replaced with the maximal number of cycles (= 40) [20]. The non-human external control (ath-miRNA 159a) differed between the serum samples with Cq values ranging between 23.0 and 26.9 (mean Cq value 24.4; SD 0.96). In previous studies using the same protocol, variation in spike-in controls has been reported to be similar [21].

TaqMan miRNA array output data (sds files) were uploaded in the ThermoFisher Cloud App (https://www.thermofisher.com/mysso/loginDisplay) and analyzed using defined threshold settings for each individual miRNA. Cq values were exported and filtered for poor amplification performance (Amplification Status, Amp Score and Cq Conf, Supplementary S1 Table). MiRNAs with average Cq values below 37 were included for statistical analysis. In addition, miRNAs were selected which had Cq values <37 in all samples, representing abundant miRNAs.

### Software and statistics

In order to correct for differences in input, global normalization was performed (Supplementary S1 Table) using QbasePlus (Biogazelle N.V., Zwijnaarde, Belgium) according to Mestdagh *et al*. [22]. A second method for normalization, also using endogenous controls, was performed. Based on an established algorithm for stability analysis (Normfinder), miRNA 93 and miRNA 130a turned out to be the most stable combination of miRNAs with mean Cq values (±SD) of 28.3 (±0.58) and 30.5 (±0.6) respectively [23](23). Normalization was also performed using the mean of these normalizers. Heatmaps of miRNA data were generated in R using the “pheatmap” clustering software package using the default settings. The Wilcoxon signed rank test was used for paired comparison of miRNA levels (ΔCq) and thyroid function tests on and off LT4 treatment. Multiple comparison adjustment was applied using the FDR approach [24].

## Results

Characteristics of 13 study patients are shown in Table 1. Mean age was 44.0 years (±SD 11.1) and mean BMI was 30.0 kg/m2 (±SD 6.0). Three of the patients (subjects 11–13) were treated with remnant ablation RAI therapy 4 weeks after thyroidectomy while the others were prepared for an extra RAI treatment by thyroid hormone withdrawal because of (suspicion of) residual or recurrent disease or positive anti-thyroglobulin (anti-Tg) antibodies.

**Table 1:** Baseline characteristics.

Subject	Age (years)	Sex	BMI (kg/m^2^)	Type of tumor	Comorbidity	Number of RAI treatments	Tg-off (μg/l)	Post-therapy I^131^ scan
1	49	Male	25.8	PTC	Pulmonary embolism	1	3.9	No uptake
2	51	Female	34.1	PTC	none	1	2.1	No uptake
3	34	Male	36.4	PTC	none	4	517.0	Sacral metastasis
4	47	Female	32.9	PTC	hypoparathyroidism	1	94.4	No uptake
5	50	Male	24.5	PTC	none	3	5.6	LN
6	20	Female	20.7	PTC	hypoparathyroidism	1	<0.9*	No uptake
7	37	Female	24.6	PTC	none	2	21.8	Mediastinal LN
8	39	Male	40.5	PTC	none	3	12.9	No uptake
9	58	Female	29.0	FTC	none	1	<0.9	Not performed
10	61	Female	26.7	PTC	hypertension	1	21.6	No uptake
11	48	Female	35.1	PTC	hypertension	0	<0.9*	Thyroid remnant
12	45	Male	34.9	PTC	hypertension	0	7.5	Thyroid remnant
13	33	Male	25.0	PTC	none	0	<0.9*	Thyroid remnant

PTC, papillary thyroid carcinoma; FTC, follicular thyroid carcinoma; BMI, body mass index; RAI, radioactive iodine; Tg, thyroglobulin; LN, lymph node.

*anti-Tg positive

As expected, thyroid function tests were significantly different on and off LT4 treatment (Table 2), reaching low levels of total T3 and FT4 with elevated TSH levels off LT4 replacement.

**Table 2:** Thyroid function tests.

	Off LT4		On LT4		*P*
TSH (0.4–4.3 mU/l)	88.9	(56.5–118.5)	0.006	(0.004–0.015)	0.001
Total T3 (1.4–2.5 nmol/l)	0.64	(0.58–0.70)	2.13	(2.0–2.3)	0.001
Free T4 (11.0–25.0 pmol/l)	1.6	(0.4–1.8)	25.7	(22.4–29.3)	0.001
Dosage LT4 (μg/kg)			2.1	(1.9–2.6)	
Time between tests, weeks (range)			24.7	(11.0–38.8)	

Changes in thyroid function tests (normal range) off and on LT4 treatment. Data are presented as median with interquartile range. LT4, levothyroxine; TSH, thyroid stimulating hormone; T3, triiodothyronine.

### Profiling of miRNAs

**Fig 1:**
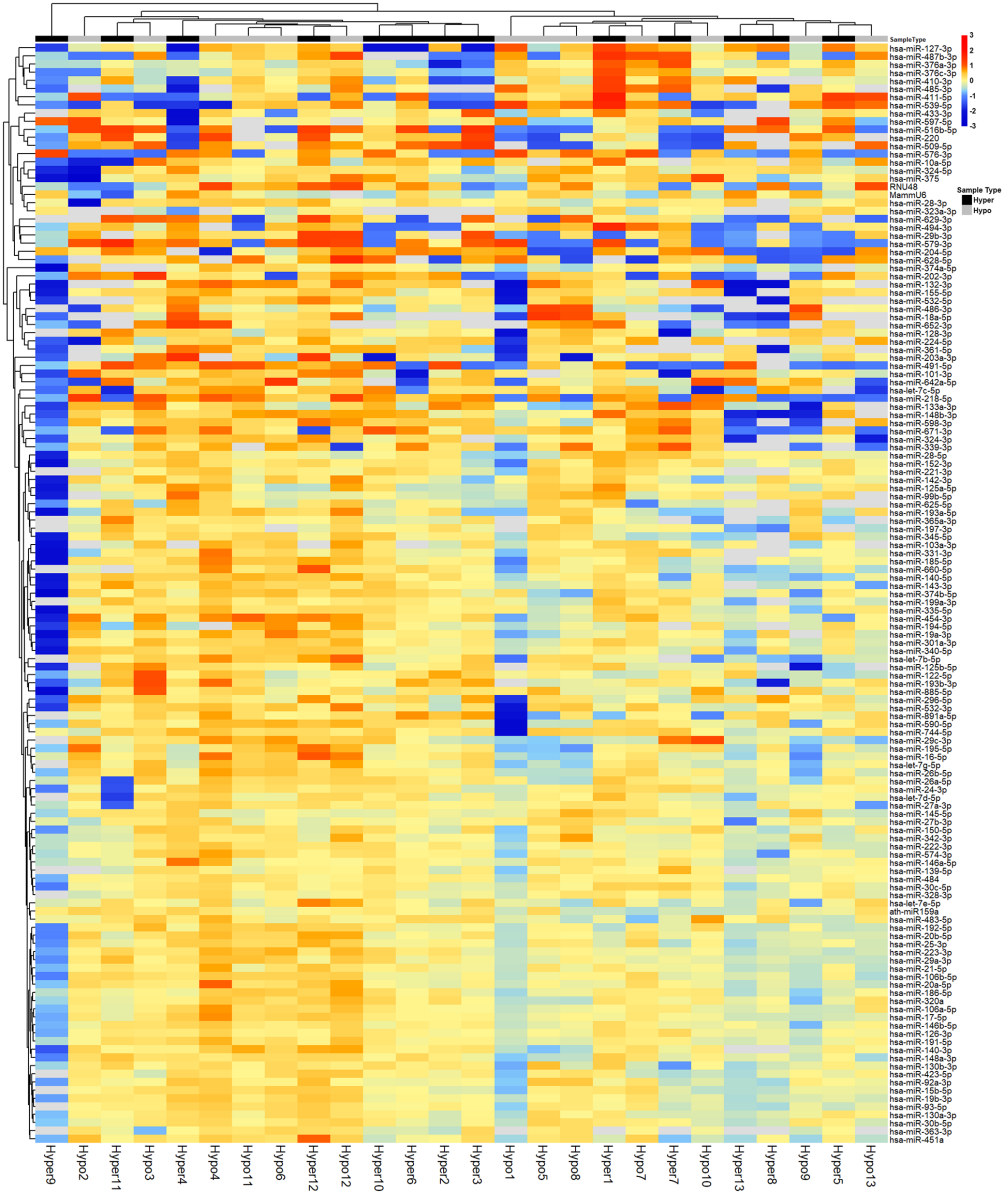
Heat map showing the Cq values of all detectable MiRNAs (n = 135) in the different thyroid states. Clustering did not group the samples according to thyroid state.

Out of the 384 miRNAs analyzed, almost half showed very low to undetectable levels. After removing all miRNAs with average Cq values > 37, only 135 miRNAs remained for analysis (S1 Table). Clustering of these globally normalized miRNAs did not group the samples according their thyroid state (Fig 1). The clustering of the abundant miRNAs (Cq values <37 in all samples, n = 59) did not separate the samples according their thyroid state as well (S1 Fig). After calibration and normalization on the global mean, the statistical analysis revealed that none of the miRNAs was significantly altered between different thyroid states after correction for multiple testing (S1 Table). As a complementary approach, we normalized the dataset on the mean levels of two endogenous reference miRNAs (miRNA 93 and miRNA 130a). This confirmed the absence of any significant differences in levels of any of the miRNAs analyzed between thyroid states (S1 Table).

#### Previously reported miRNAs

The profiles of miRNAs which have been previously reported to be differentially expressed in muscle and liver in hypothyroidism were evaluated [13, 14]. The level of miRNA 1 and miRNA 133b were not amongst the 135 detectable miRNAs (S1 Table). MiRNA 206 was not on the card. MiRNA 133a showed a mean Cq value of 31.2 (±1.2) in hypothyroid state versus 31.3 (±1.7) in hyperthyroid state (not significantly different).

## Discussion

To our knowledge, this is the first study investigating a selected multi-target miRNA profile in serum of individual patients during (extreme) changes in thyroid states. Most studies on miRNAs in patients with thyroid disease have focused on thyroid cancer [25]. A study by Yamada *et al*, reported several miRNA levels that were differently expressed in serum from patients with autoimmune thyroid disease compared with healthy subjects [26]. They suggested that the underlying autoimmune condition was responsible for the observed changes since there was no association between the mentioned miRNAs and TSH levels. However they did not study the changes in miRNA expression after restoring euthyroidism [26]. Another study demonstrated that different levels of circulating miRNAs are associated with intractable Graves’ disease compared with Graves’ disease in remission [27].

In our study, we could not find significant changes in levels of serum miRNAs in athyroid hypothyroid patients on and off LT4 treatment, despite previous studies showing clear differences in (precursors of) miRNAs in different tissues [13, 14, 28]. There are a number of possible explanations for our results. First, our sample size might have been too small to discover significant changes in miRNA levels. However, previously, in a cohort of similar size, microarray analysis revealed large differences between thyroid states in muscle samples, suggesting that the used sample size was sufficient if the differences would have been equally large and consistent [13]. McDonald *et al*. found that assay imprecision (due to variability in the RNA extraction process and interassay imprecision) had significant effects on the reproducibility of miRNA measurements and concluded that only miRNAs that are extremely up- or downregulated will be suitable as clinical biomarkers [29]. If there were such extremely up- or downregulated miRNAs they should have been detected in our sample size. Second, although it has been shown that miRNAs are present in human plasma and serum in a remarkably stable form that is protected from endogenous RNase activity [15], differences in the time between blood drawing and storage potentially impact on the miRNA levels released from blood cells, in particular if samples are stored over 24h [29]. However, all our samples were stored at -80 °C within 2 to 4 hours of collection and therefore a significant effect on miRNA expression is not likely [30]. Third, although after calibration, normalization of the data was performed using two different methods (based on the global mean and based on mean levels of two endogenous reference miRNAs) providing similar results, we cannot rule out that a variation in protocol efficiency might have influenced our results. Finally, although several studies have shown that thyroid hormones seem to regulate miRNA expression in several organs such as skeletal muscle, liver and heart, these changes in tissue miRNA expression may not be reflected in serum [13, 14, 28]. It has to be mentioned that our previous study in muscle samples examined only precursors of mature miRNAs (pri-miRNAs) while our current study investigated the effects of TH on the levels of mature miRNAs. However, Dong *et al*. found significant decreases of both mature miRNAs and it’s corresponding precursor miRNAs in liver samples of hypothyroid mice compared to euthyroid controls [14].

Our study has several strengths and limitations. A limitation of our study is that we only studied a selection of 384 miRNAs and we cannot rule out that other miRNAs, which were not included in our platform are influenced by thyroid state. For example, miRNA 206, of which the precursor pri-miRNA-206 differed significantly in skeletal muscle, was not included in the current platform. A possible confounder of our study is that we studied patients with DTC, while several miRNAs in serum have been postulated as biomarker for DTC [25]. The levels of serum let-7e, miRNA-151-5p, microRNA-146b and miRNA-222 have been reported to be significantly increased in PTC patients relative to healthy controls [31, 32]. Circulating levels of miRNA-146a-5p, miRNA-146b-5p, miRNA-221-3p, and miRNA-222-3p have been shown to decline after tumor excision [33]. Although seven of our patients did not show any uptake on the post-therapy RAI scan and Tg-off levels were mostly low, some miRNAs theoretically could have been changed by the response of the tumor to RAI therapy or to the radiation it selves. Finally, although all patients with active inflammatory disease or other malignancies were excluded, some patients had comorbidities which might have affected their T3-dependent miRNA profile. Our study has several strengths as well. First, the study design included paired analyses, which has the advantage to reduce confounders. Second, we were able to study extreme differences in thyroid state in human subjects. Finally, we used a validated and widely used method to generate the serum miRNA profile [20, 34–36].

In conclusion, although we previously showed regulation of pri-miRNAs by thyroid hormone in muscle samples, the present study using an extensive panel of 384 miRNAs does not supply evidence of regulation of mature miRNAs in serum by thyroid hormone.

## Supporting information

S1 Table Raw data and results of the statistical analysis with normalization on the global mean. (XLSX)

S1 Fig Heat map showing the Cq values of the abundant miRNAs (Cq values <37 in all samples, n = 59) in the different thyroid states. Clustering did not group the samples according to thyroid state.
(PDF)
