# Supplementary figures and images for: Serum microRNA profiles in athyroid patients on and off levothyroxine therapy
Source: PLoS One. 2018 Apr 12;13(4):e0194259. doi: 10.1371/journal.pone.0194259 (PMC5896904; doi:10.1371/journal.pone.0194259)

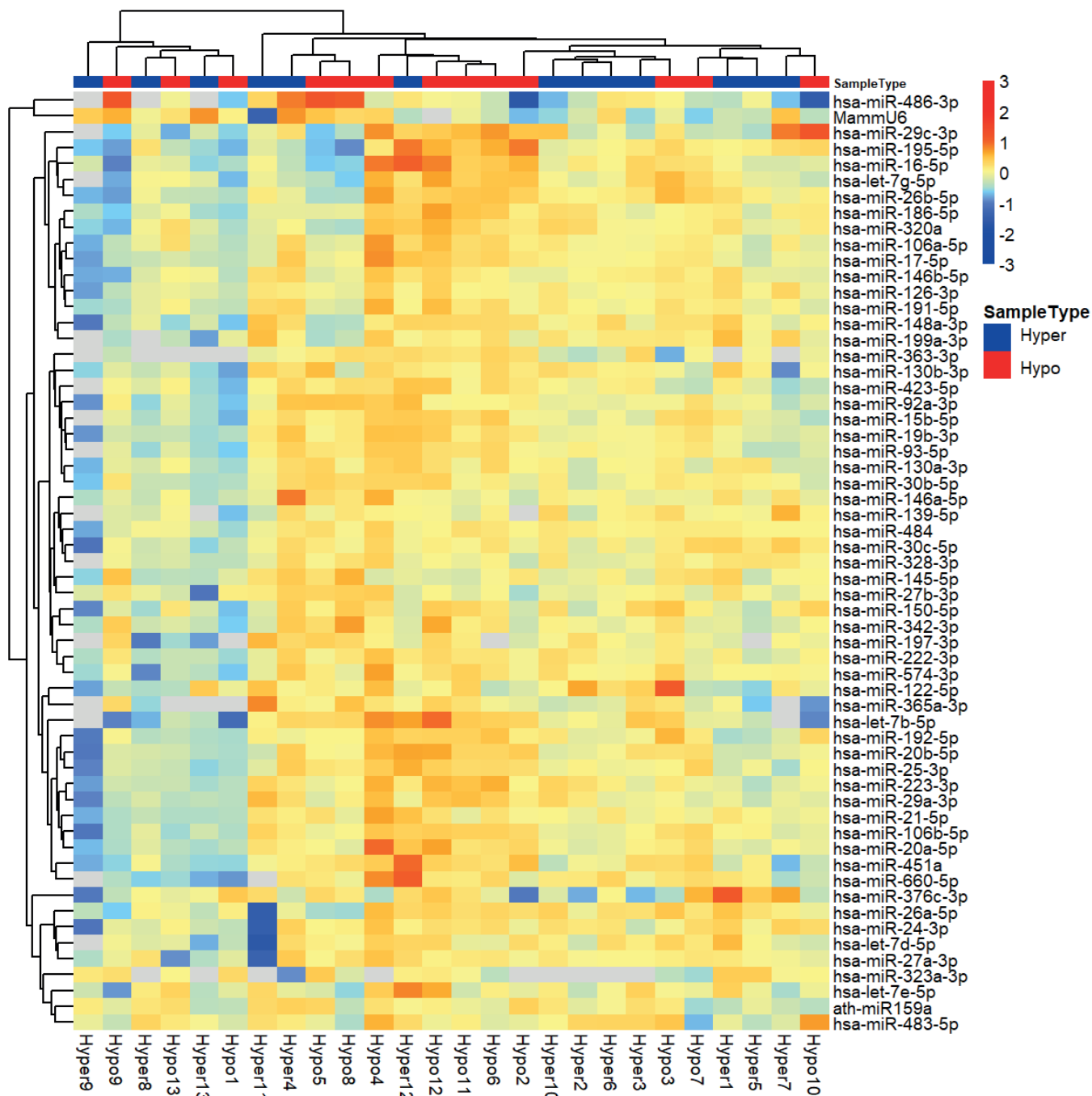

Supplement: S1 Fig — Clustering did not group the samples according to thyroid state. (PDF) [file pone.0194259.s002.pdf]
